# Blackcurrant (*Ribes nigrum*) lowers sugar-induced postprandial glycaemia independently and in a product with fermented quinoa: a randomised crossover trial

**DOI:** 10.1017/S0007114520004468

**Published:** 2021-09-14

**Authors:** Jenni Lappi, Kaisa Raninen, Kati Väkeväinen, Anna Kårlund, Riitta Törrönen, Marjukka Kolehmainen

**Affiliations:** 1 Faculty of Business, Tourism and Hospitality, Savonia University of Applied Sciences, 70201 Kuopio, Finland; 2 Institute of Public Health and Clinical Nutrition, University of Eastern Finland, 70211 Kuopio, Finland; 3 SIB Labs Infrastructural Unit, University of Eastern Finland, 70211 Kuopio, Finland

**Keywords:** Blackcurrants, Fermented quinoa, Postprandial metabolism, Glycaemia, Food products

## Abstract

Berries rich in anthocyanins have beneficial effects on postprandial glycaemia. We investigated whether blackcurrant (75 g in a portion) independently and in a product with fermented quinoa induced similar effects on the sugar-induced postprandial glucose metabolism as observed before with 150 g of blackcurrant. Twenty-six healthy subjects (twenty-two females and four males) consumed four test products after fasting overnight in a randomised, controlled crossover design. Each test product portion contained 31 g of available carbohydrates and had similar composition of sugar components: 300 ml water with sucrose, glucose and fructose (SW; reference), blackcurrant purée with added sugars (BC), a product consisting of the blackcurrant purée and a product base with fermented quinoa (BCP) and the product base without blackcurrant (PB). Blood samples were collected at 0, 15, 30, 45, 60, 90, 120 and 180 min after eating each test product to analyse the concentrations of glucose, insulin and NEFA. In comparison with the SW, the intake of both the BC and BCP resulted in reduced glucose and insulin concentrations during the first 30 min, a more balanced decline during the first hour and improved glycaemic profile. The BCP induced more efficient effects than the BC due to the product base with fermented quinoa. A rebound of NEFA after the sugar-induced hypoglycaemic response was attenuated at the late postprandial phase by the BC and BCP. In conclusion, we showed that 75 g of blackcurrant and the product with fermented quinoa were able to lower postprandial glycaemia and insulinaemia.

## Graphical abstract

**Figure:**
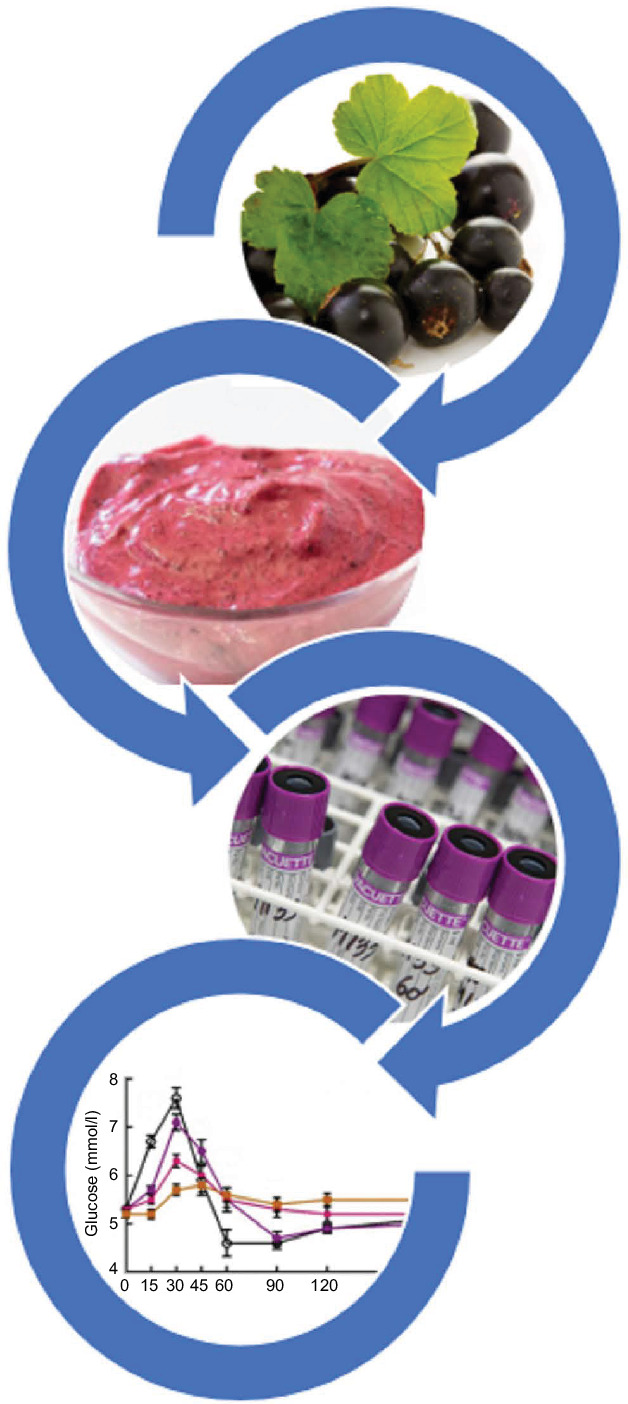

Berries are an important component of a healthy Nordic diet^(1)^ that supports the prevention of non-communicable diseases, such as CVD^(2)^ and type 2 diabetes^(3)^. High-carbohydrate, low-fibre meals rapidly increase blood glucose and insulin levels, and for a few postprandial hours, expose the body to oxidative stress and inflammatory challenges^(4,5)^. If recurring regularly during a long period of time, large variations in the blood glucose and insulin levels may increase the risk factors of the metabolic syndrome, by increasing hunger and eating, reducing the insulin sensitivity of tissues, activating the immune system for low-grade inflammation^(6,7)^ and causing changes in the normal function of blood vessels^(8)^.

Berries have been found to have beneficial effects on the postprandial glucose metabolism^(9–16)^. These effects were associated with berry-derived polyphenolic compounds, especially anthocyanins rich in dark-coloured berries^(17)^. Polyphenolic compounds may slow down the absorption of glucose from the small intestine, by interacting with carbohydrate-digesting enzymes and glucose transport proteins^(18,19)^. In addition, polyphenolic compounds may reduce oxidative stress^(20)^ and inflammation^(21)^. In previous short- and long-term intervention studies, dark berries, in general, have been partly but not consistently associated with reduced low-grade inflammation measured by biomarkers such as various IL and high-sensitive C-reactive protein (hs-CRP)^(7,22–24)^.

In comparison with many other berries, the blackcurrant is rich in anthocyanins^(25)^ and easy to cultivate, thus making it an interesting raw material for the food industry and health-conscious consumers. Although the nutritional value of blackcurrants is high, blackcurrant is currently not utilised efficiently in food products. Consumers may find its natural sourness, bitterness and astringency unpleasant, unless an adequate amount of sugar is added^(26,27)^. Thus, there is a great need for new, innovative berry products with high nutritional and sensory quality, and with high potential for increased health-related functionalities. In our earlier studies showing favourable effects on the postprandial glucose metabolism, a 150 g portion of purée made of whole blackcurrants was used^(11,13)^. However, from the food industry and consumer points-of-view, using as high as 150 g of blackcurrants in one portion sets challenges for the development of pleasant and palatable commercial berry products. Thus, we halved this amount in the present study since 75 g of blackcurrant is more suitable for the development of diverse berry products for one portion.

Quinoa is an interesting raw material because it is naturally gluten-free and has high-fibre and protein contents. In general, the native taste and texture of cereal-based products can be technologically modified to more pleasant attributes by using fermentation^(28)^. Quinoa-based ingredients are well suited for fermented food applications of high sensory and nutritional quality^(29)^. Some human interventions have indicated that quinoa may have a modest improving effect on blood lipid profile and glucose metabolism in elderly or obese subjects^(30,31)^. Still, a postprandial study showed no difference in glycaemic response between ingested quinoa and white wheat bread with equal amounts of available carbohydrates^(32)^. Although fermented quinoa has no confirmed health effects, in combination with berries and fruits, quinoa-based fermented products are perceived as healthy by the consumers^(29)^. Thus, fermented quinoa is a nutritious and well-fitted matrix for palatable snack products.

The aim of the present study was to gain more scientific knowledge on the effect of whole blackcurrants ingested as a 75 g portion, as well as when incorporated in a newly produced palatable snack product with fermented quinoa, on the postprandial glucose metabolism, induced by intake of a reasonable amount of sugar. Primary outcomes in the study were incremental glucose and insulin peaks, area under the glucose and insulin curves, and glycaemic profile (GP), which were derived from blood glucose and insulin concentrations measured at 0–180 min. Secondary, we aimed to investigate effects on postprandial NEFA and hs-CRP. Hs-CRP was chosen for biomarker of inflammation because it has been shown to respond to oral glucose load after 1 h of the load as reviewed by Calder *et al.* ^(33)^. Based on the previous studies, we hypothesised that the ingested 75 g of blackcurrant is able to attenuate the increase of glucose and insulin concentrations in the early postprandial phase and to retard the decrease of the glucose concentration below the fasting level. We also hypothesised that these effects will remain when the same amount of blackcurrant is ingested in a newly developed snack product, in which blackcurrant was mixed with a product base (PB) containing fermented quinoa.

## Methods

### Participants

Participants for the study were recruited by an announcement in a local newspaper and on noticeboards at the local universities’ campus areas and intranet, as well as via social media of a food sector network Food Valley in Eastern Finland. The inclusion criteria for the participants were as follows: healthy males or females aged 25–65 years with normal weight or slightly overweight (BMI 20–28 kg/m^2^), with no diabetes or other chronic diseases affecting the glucose metabolism, no smoking, no celiac disease and no pregnancy or lactating. In addition, low-carbohydrate or vegan diet or weight gain or loss >5 % over the past 6 months was not allowed. Drinking wine or spirits was restricted to less than a portion of 120 or 40 ml, respectively, three times per week, and drinking beer and cider was restricted to less than daily. Regarding food supplements, possible use was asked to remain constant over the study. Attending other clinical trials or blood-sample donations shortly before or concurrently with the present study was not allowed.

**Fig. 1.**
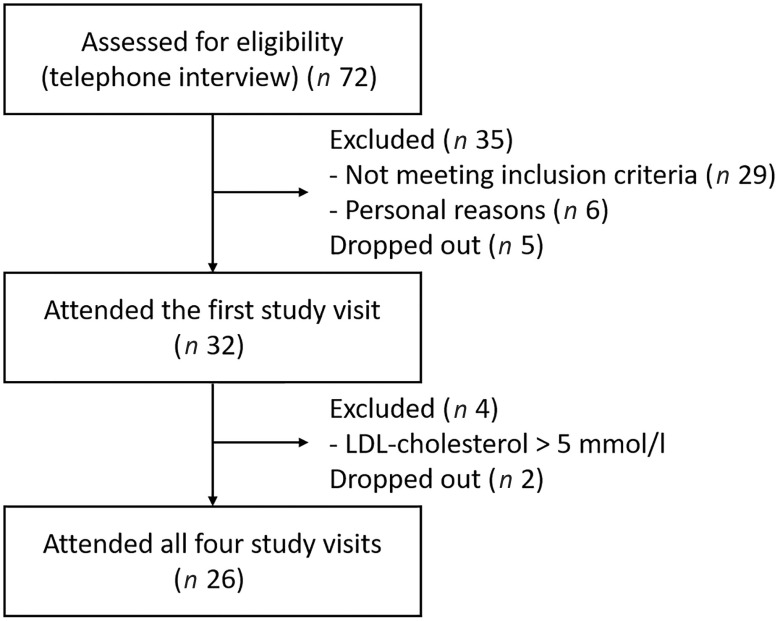
Flow diagram of the participant recruitment.

On the recruitment, the participants contacted the study nurse, who shortly interviewed them via telephone to preliminary assess whether they fit the inclusion criteria (Fig. 1). The participants’ health status and medical history were assessed using a structured form and by anthropometric and laboratory measurements (fasting plasma glucose and lipids, thyroid-stimulating hormone, alanine aminotransferase and creatinine). If these measures indicated exclusion criteria, the participants were excluded from the study after the first study visit.

The present study was conducted according to the guidelines laid down in the Declaration of Helsinki and all procedures involving human participants were approved by the Research Ethics Committee of the Northern Savo Hospital District (Finland) (894/2019). Written informed consent was obtained from all participants. The study was registered at clinicaltrials.gov as NCT04150926.

### Test products

The four test products each contained 31 g of available carbohydrates and were sugary water (SW), blackcurrant purée (BC), a blackcurrant product (BCP) and a fermented quinoa PB (Table 1). Since the amount of available carbohydrates was matched for each test product to investigate the postprandial glycaemic effects, the portion sizes differed as follows: 300 ml for the liquid SW, and 100, 147 and 78 g for the solid BC, BCP and PB, respectively. The test products had similar composition of sugar components per portion. The sugar composition in each product was adjusted according to the amounts of added sucrose and the concentrations of natural sucrose, glucose and fructose in the BC. The most pleasant amount of the added sucrose (25·5 g) with 75 g of BC was chosen as a result of a previous sensory evaluation made by a consumer panel (results not shown). For the BCP and the PB, 2·4 g less sucrose per portion was added to balance with the amount of starch originating from the fermented quinoa, thus matching the amount of available carbohydrates in each test product portion. As for macronutrients, the fat content of the SW, BC, BCP and PB was 0, 0·2, 10 and 10 g, respectively (Table 1). The protein content of the SW, BC, BCP and PB was 0, 0·9, 1·0 and 0·3 g and that of total fibre 0, 5·2, 10·6 and 5·4 g, respectively.

**Table 1.** Nutrient composition, acidity and viscosity of the test products

	SW (300 ml)	BC (100 g)	BCP (147 g)	PB (78 g)
Fat (g)* †	0	0·2	10	10
Available carbohydrates (g)‡	31	31	31	31
Sucrose (g)	25·5	25·5	23·1	23·1
Glucose (g)	2·3	2·3	2·3	2·3
Fructose (g)	2·8	2·8	2·8	2·8
Starch (g)	0	0	2·4	2·4
Protein (g)†	0	0·9	1·0	0·3
Total fibre (g)§	0	5·2	10·6	5·4
Anthocyanins (mg)†	0	187	187	0
Proanthocyanidins (mg)†	0	263	263	0
pH†	–	3·0	3·1	4·0
TTA (ml of NaOH)†	–	24·7	37·7	0·4
Viscosity (Pas)†	–	34	85	–

SW, sugary water; BC, blackcurrant purée; BCP, blackcurrant product; PB, product base; TTA, titratable acidity.

* Analysed value for BC and calculated for BCP and PB based on content of fat in the ingredients used.

† The results are the means of triplicates.

‡ Calculated from analysed amounts of native sugars and starch in ingredients and from the added sucrose.

§ Analysed value for BC and calculated for BCP and PB based on the analysis in BC and calculated content of fibre in the ingredients.

The SW was prepared by adding 25·5, 2·8 and 2·3 g of sucrose, fructose and glucose, respectively, to 300 ml water. The SW was stored at 5°C until being served to the participants. The other test products were prepared in advance in batches from which weighed portions were stored as frozen (−18°C), until being thawed overnight at 5°C for serving to the participants.

Frozen blackcurrants (*Ribes nigrum* (Öjebyn)) of Finnish origin were provided by Pakkasmarja Ltd. Whole blackcurrants were mashed by an industrial colloid mill (PVS Systemtechnik GmbH), using a blade with diameter <0·35 mm, to a fine-grained purée. The purée was analysed in triplicate according to previously published methods: the sugars and anthocyanins were determined in their native forms by the HPLC refractive index detector^(34)^ and HPLC with diode-array detection^(13)^, respectively. The proanthocyanidins were determined by HPLC-diode-array detection and fluorescence detection after thiolytic degradation^(35)^. To calculate the amount of total anthocyanins, the concentrations of following were summed up: delphinidin 3-*O*-glucoside, delphinidin 3-*O*-rutinoside, cyanidin 3-*O*-glucoside, cyanidin 3-*O*-rutinoside, petunidin 3-*O*-rutinoside, peonidin 3-*O*-rutinoside, cyanidin 3-*O*-(6″-p-coumaroyl-glucoside) and petunidin 3-*O*-(6″-p-coumaroyl-glucoside).

The BC was prepared by adding 25 g of sucrose to 75 g of semi-frozen fine-grained BC and mixing with a cutter (Robot coupe R45). The BCP was prepared by utilising a fermented quinoa base, sugar, rapeseed oil and stabilising additives to prepare a PB to which 75 g of the BC was added. For the quinoa base, only little quinoa was used: 3 g of quinoa flour per the final portions of the BCP and PB was mixed with water, and fermented until pH < 4·0 by *Lactobacillus plantarum* Q823, a starter culture previously used to prepare quinoa products^(29)^ and isolated from quinoa^(36)^. The fermented quinoa base was cooled down to 6°C and other ingredients were added: sucrose, rapeseed oil, water, *α*-cyclodextrin (Wacker Chemie AG), guar gum and locust bean gum (Unipektin Ingredients AG). The proportion of the fermented quinoa base in the final BCP was 17·7 %. The PB was prepared as described, but the BC was left out and 0·5, 2·3 and 2·8 g more sucrose, glucose and fructose were added per portion, respectively, to compensate for the amount of natural blackcurrant sugars in the BCP.

The fermented quinoa base was freeze-dried and analysed for native starch (enzymatic UV-method, catalogue no. 10 207 748 035, R-Biopharm AG) and sugars (HPLC with pulsed amperometric detector).

The nutritional compositions (fat for BC and protein for BC, BCP and PB) were analysed using standard methods of the Association of Official Analytical Chemists (AOAC)^(37)^ in triplicates (Table 1). In addition, the BC was analysed for total fibre and inulin by standard enzymatic-gravimetric and enzymatic-photometric methods (Association of Official Analytical Chemists 985.29 and ASU L 00.00-94, respectively). The pH, total titratable acidity and viscosity (spindle 7, Rotary Viscometer PCR-RVI3, 127 Model 20) of the products were also measured^(29)^.

### Design

The study was a randomised, controlled crossover postprandial study with four separate study visits at least 5 d apart. Before the study started, an investigator assigned the participants to eat the test products in random order by using the function ‘Random’ in Excel. Before the first study visit, the participants were instructed to consume an evening meal of their choice. Before the other three visits, they were asked to eat a similar meal at the same time in the preceding evening. The evening meals were recorded by the participants and the records checked by a research assistant at every visit. Furthermore, the participants were asked to keep their diet, body weight and living habits constant during the study, to refrain from intensive physical activity for 12 h before the test, and not to consume any berries or berry products the day before.

Each test visit was conducted with the following procedure: the tests were carried out in the morning after 10–12 h of overnight fasting. The study nurse interviewed the participants on their health status and measured their blood pressure and body weight. For the collection of venous blood samples, an intravenous catheter was inserted in an antecubital vein of the forearm. After this, the participants were offered the test product and asked to consume each product in less than 10 min. The eating time was recorded. Baseline blood samples were drawn at the fasting state (0 min), and the other samples at 15, 30, 45, 60, 90, 120 and 180 min after starting to consume the test product. The samples for plasma glucose measurements were collected in citrate-fluoride tubes, for plasma insulin and NEFA measurements in EDTA tubes kept on ice and for plasma hs-CRP measurements in lithium-heparin tubes. Plasma was immediately separated by centrifugation at 4°C and stored at −70°C.

### Laboratory analyses

The plasma glucose concentrations were analysed with the hexokinase method using reagents for cobas c systems (glucose HK, Roche Diagnostics GmbH) and a cobas 8000 c702 analyzer (Hitachi High Technology Co.). The plasma insulin was measured with a chemiluminometric immunoassay using a Liaison analyzer (DiaSorin Deutschland GmbH). The plasma NEFA concentrations were measured with an enzymatic colorimetric method using Wako NEFA-HR(2) reagents (Wako Chemicals GmbH) and a Konelab 20 XTi analyzer. For analysing the hs-CRP, a particle-enhanced immunoturbidimetric method with reagents for cobas c systems (C-reactive protein (Latex) high sensitive assay, Roche Diagnostics GmbH) and a cobas 8000 c502 analyzer (Hitachi High Technology Co.) were used.

For screening of the general health status, the following methodology was used: the total cholesterol, TAG and creatinine were analysed with an enzymatic colorimetric method using reagents for the cobas c systems (Cholesterol Gen.2, TAG and Creatinine plus version 2, respectively, Roche Diagnostics GmbH) and the cobas 8000 c702 analyzer (Hitachi High Technology Co.). The same analyzer was used to measure the LDL-cholesterol and the HDL-cholesterol with a homogeneous enzymatic colorimetric assay and alanine aminotransferase with a kinetic method according to the International Federation of Clinical Chemistry and Laboratory Medicine, using reagents for the cobas c systems (LDL-Cholesterol Gen.3, HDL-Cholesterol Gen.4, Alanine Aminotransferase, International Federation of Clinical Chemistry and Laboratory Medicine with pyridoxal phosphate, respectively, Roche Diagnostics GmbH). The thyroid-stimulating hormone levels were analysed with an electrochemiluminescence immunoassay using a cobas e801 analyzer (Hitachi High Technology Co.).

### Calculations and statistical analyses

The data were analysed using IBM SPSS Statistics for Windows, version 25.0 (IMB Corp.) and are expressed as means and standard deviations or standard errors of the mean as described in text, tables and figures. Sufficient sample size was evaluated based on previous studies^(11,13)^, assuming 80 % power and the *α* (*P*) level of 5 % (two-sided testing). For observing statistically significant differences in the incremental glucose peaks, the areas under the glucose curves and the GP, the calculated sample sizes were 12–14, 26–34 and 6, respectively. Thus, our aim was to recruit thirty participants, allowing for 15 % drop-out rate.

Linear mixed-effect modelling was used to compare the effects of the test products with adjustment (Sidak). Histograms were used for checking the normality of the model residuals, and logarithmic transformation of variables was used if the normality was improved, that is, for the GP (illustrating the form of the glucose curve), glucose and insulin AUC and the incremental peak of insulin. Values of *P* < 0·05 were considered as statistically significant. The statistical significance of the product × time interaction was tested in the mixed-model analysis using participants as a random factor and product × time and their main effects as fixed factors. When the product × time interaction was statistically significant, the differences between the test products at individual time points were tested by *post hoc* analysis with Sidak adjustment for multiple comparisons. For monitoring the possible effect of sex, the statistical analyses were re-examined using sex as a covariate in the model, as well as excluding males. As no impact regarding these was observed in the results, the final analyses were performed with all the subjects without adjustment for sex.

The incremental peak of glucose and insulin values was calculated by subtracting the fasting value from the highest value. The GP was calculated for each participant and test product by dividing the time (min) during which the plasma glucose was above the fasting concentration with the incremental peak glucose value (mmol/l)^(38)^, using GraphPad Prism 5.03 for Windows software (GraphPad Software Inc.). The incremental AUC was calculated using the GraphPad Prism software, ignoring the area below the fasting (0 min) concentration. The differences in incremental peaks, GP, AUCs, eating times of the products and weight of the subjects among and between the test products or visits were tested using the participant as a random factor and the product as a fixed factor in the linear mixed-effect model analysis. In the hs-CRP, fourteen individuals had values under the detection limit of 0·15 mmol/l. These were calculated as 0·14 mmol/l in the data analysis.

## Results

### Participant characteristics

Twenty-six participants fulfilled the inclusion criteria and conducted the whole postprandial study with the four study visits (Fig. 1). The basic characteristics of the participants are presented in Table 2. The weight of the participants remained stable throughout the study (*P* = 0·143). The eating times of the four test products differed statistically (*P* < 0·001) and were 5·0 (sd 0·7) min for SW, 4·9 (sd 0·8) min for BC and 5·1 (sd 0·8) min for BCP and PB.

**Table 2.** Basic characteristics of the participants (*n* 26; twenty-two females, four males) (Mean values and standard deviations)

	Mean	sd
Age (years)	50	15
Weight (kg)	68	11
BMI (kg/m^2^)	24	3
Waist circumference (cm)		
Female	79	7
Male	96	12
Blood pressure (mmHg)		
Diastolic	82	9
Systolic	127	18
Fasting plasma glucose (mmol/l)	5·4	0·4
Fasting plasma total cholesterol (mmol/l)	4·7	0·9
Fasting plasma LDL-cholesterol (mmol/l)	3·0	0·8
Fasting plasma HDL-cholesterol (mmol/l)	1·7	0·3
Fasting plasma TAG (mmol/l)	1·0	0·5

### Glucose, insulin and NEFA

**Fig. 2.**
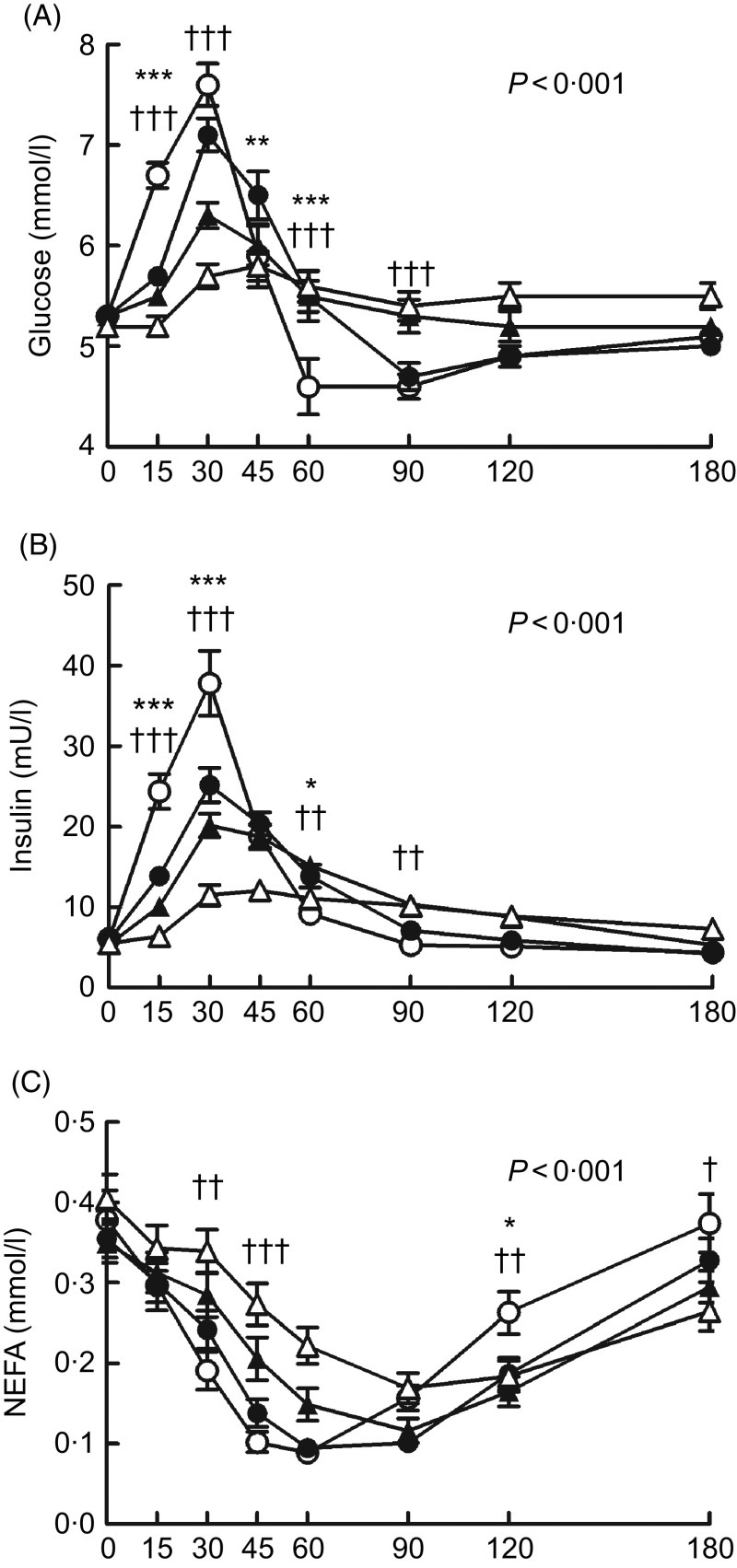
The plasma glucose (A), insulin (B) and NEFA (C) concentrations after ingestion of the test products: sugary water (○), blackcurrant purée (●), blackcurrant product (▲) and product base (Δ). The values are means with their standard errors (*n* 26). The *P* values in the figures indicate the level of significance of time × product interactions in the linear mixed-effect model. The blackcurrant purée was significantly different from that of the sugary water at an individual time point: * *P* < 0·05, ** *P* < 0·01, *** *P* < 0·001 (*post hoc* analysis with Sidak adjustment). The blackcurrant product was significantly different from that of the sugary water at an individual time point: † *P* < 0·05, †† *P* < 0·01, ††† *P* < 0·001 (*post hoc* analysis with Sidak adjustment).

The postprandial glucose responses (Fig. 2(A)) differed among the test products (*P* < 0·001 for product × time interaction). The GP was more favourable after the BC, BCP and PB as compared with the SW (Table 3). The plasma glucose concentration increased more moderately after the BC as compared with the SW and was significantly lower at 15 min (*P* < 0·001) and slightly, but not significantly, lower at 30 min (*P* = 0·055), and higher at 45 min (*P* < 0·05) and 60 min (*P* < 0·001). The BCP lowered and balanced the glycaemic response even more; the postprandial glucose was lower at 15 and 30 min (*P* < 0·001 in both) as compared with the SW and also avoided dramatic dropping under the fasting level, being significantly higher at 60 and 90 min (*P* < 0·001 in both). The glucose concentration for the BCP as compared with the BC was significantly lower at 30 min (*P* < 0·001) and 45 min (*P* < 0·05), and higher at 90 min (*P* < 0·01). The PB had also an independent lowering and balancing effect for the glycaemic response; when the BCP was compared with the PB, the glucose concentration was lower with the PB at 30 min (*P* < 0·01). The incremental glucose peak was lowered by 17 % (*P* < 0·05), 50 % (*P* < 0·001) and 67 % (*P* < 0·001) for the BC, BCP and PB, respectively, as compared with SW (Table 3).

**Table 3.** Glucose and insulin variables after the intake of the test products (*n* 26)† (Mean values and standard deviations)

	SW		BC		BCP		PB		*P* ‡
	Mean	sd	Mean	sd	Mean	sd	Mean	sd	
Glycaemic profile (min/(mmol/l))	23·9	6·8	41·8**	18·5	74·2***	34·1	204·4***	186·9	<0·001
Incremental glucose peak (mmol/l)	2·4	1·0	2·0*	0·8	1·2***	0·6	0·8***	0·5	<0·001
Glucose AUC (mmol/l × min)	76·1	59·8	69·6	46·9	58·9	47·9	62·7*	50·3	0·031
Incremental insulin peak (mU/l)	32·0	19·1	21·0**	9·0	16·9***	5·9	9·2***	5·6	<0·001
Insulin AUC (mU/l × min)	1055·4	502·0	898·0	406·3	1050·2	453·5	702·7***	389·6	<0·001

SW, sugary water; BC, blackcurrant purée; BCP, blackcurrant product; PB, product base.

Mean value was significantly different from that of the SW: * *P* < 0·05, ** *P* < 0·01, *** *P* < 0·001 (*post hoc* analysis with Sidak adjustment).

† Except for the glycaemic profile for SW (*n* 25) due to a missing value necessary for the calculation of the glycaemic profile.

‡ Linear mixed-effect model analysis.

The postprandial insulin responses (Fig. 2(B)) followed the observed glucose responses and were different among the test products (*P* < 0·001 for product × time interaction). The postprandial plasma insulin concentration was significantly lower for BC, BCP and PB compared with SW at 15 and 30 min (*P* < 0·001 for all). The BCP induced a significantly lower insulin concentration than the BC at 30 min (*P* < 0·01). When the BCP was compared with the PB, the insulin concentration was lower with the PB at 30 min (*P* < 0·001), 45 min (*P* < 0·001) and 60 min (*P* < 0·05). The incremental peak of insulin was lowered by 34 % (*P* < 0·01), 47 % (*P* < 0·001) and 71 % (*P* < 0·001) for BC, BCP and PB, respectively, as compared with the SW (Table 3).

The NEFA response differed among the test products (*P* < 0·001 for product × time interaction). The plasma NEFA concentrations decreased postprandially more slowly after the BCP and PB (Fig. 2(C)), the differences between the BCP and SW being statistically significant at 30 and 45 min (*P* < 0·01, *P* < 0·001, respectively). This was not seen after the BC. The NEFA concentration for the BCP was higher than for the BC at 45 min (*P* < 0·05). Furthermore, the concentration of NEFA turned to rise earlier after ingestion of the SW as compared with the other products: NEFA for the SW was significantly higher at 120 min as compared with the BCP (*P* < 0·01) and with the BC (*P* < 0·05), and at 180 min (*P* < 0·05) as compared with the BCP.

We also measured the effect of these products on the circulating hs-CRP concentration (Table 4). The detected hs-CRP levels were low in these healthy participants, and there was no effect on acute inflammation with any of these products compared with the glucose concentration. Fourteen individuals had part of the hs-CRP values under the detection limit.

**Table 4.** Highly sensitive C-reactive protein (hs-CRP) concentrations after intake of the test products (*n* 26)* (Mean values and standard deviations)

hs-CRP (mmol/l)	SW		BC		BCP		PB		*P* †
	Mean	sd	Mean	sd	Mean	sd	Mean	sd	
0 min	0·72	0·82	0·58	0·64	0·94	1·24	0·63	0·65	NS
30 min	0·68	0·78	0·56	0·63	0·88	1·24	0·59	0·66	NS
60 min	0·69	0·84	0·57	0·64	0·88	1·22	0·51	0·63	NS
120 min	0·73	0·82	0·56	0·60	0·89	1·18	0·59	0·64	NS
180 min	0·74	0·79	0·55	0·65	0·94	1·22	0·61	0·66	NS

SW, sugary water; BC, blackcurrant purée; BCP, blackcurrant product; PB, product base.

* Fourteen individuals had part of the hs-CRP values under the detection limit of 0·15 mmol/l, these were calculated as 0·14 mmol/l in the data analysis.

† Linear mixed-effect model analysis.

## Discussion

In this clinical postprandial study, we demonstrated that 75 g of whole blackcurrants as purée was able to lower glycaemic response after eating the blackcurrants with sugar. Our results of the effect of blackcurrant on balancing the postprandial glycaemic response were similar to those observed in our previous studies^(11,13)^. Surprisingly, the new snack product containing 75 g of blackcurrant and the fermented quinoa base reinforced the lowering effect observed for blackcurrants only. The effects were shown with twenty-six healthy, mainly female participants, who consumed 31 g of available carbohydrates in four different products: a reference (SW), two products (BC and BCP) containing the same amount of blackcurrant (75 g) and a PB with fermented quinoa but without the blackcurrant (PB).

Ingestion of the SW induced a rapid and high initial elevation in glucose and insulin concentrations. Due to the high insulin response, the glucose concentrations rapidly decreased below the fasting level and reached the lowest concentration – below the fasting level – already at 1 h. The glucose concentration stayed below the fasting level until the end of the test period. The hypoglycaemic response was accompanied by a compensatory elevation in NEFA concentrations. These effects on glucose and insulin responses were attenuated when 75 g of blackcurrants in the BC was consumed with the same amount of sugar. Ingestion of the BC resulted in a more beneficial GP in line with a reduced postprandial insulin response, as compared with the SW. A higher value of the GP is considered more beneficial, since the high GP value is related to the low incremental glucose peak and the less pronounced late hypoglycaemia^(38)^.

In the present study, the observed effects were shown with half the amount of blackcurrant than in the previous studies (150 g). The proportion of blackcurrant (g) to available carbohydrates (g) was 2·5 to 1 in the present study. The same proportions were 3·2–3·4 to 1 and 4·3 to 1 in our previous studies^(11,13)^. When reflecting these proportions to the observed reductions in the incremental peak of glucose, a dose–response-like effect emerged. As compared with references, the reduction in the incremental glucose peak was 17 % for the BC whereas that was 22–30 % for a BC and nectar containing 3·2–3·4 times more blackcurrants than available carbohydrates^(11)^, and 33 % for a blackcurrant nectar containing 4·3 times more blackcurrants than available carbohydrates^(13)^. The similar dose–response-like effect can be observed for the GP: 75 % improvement in the GP in our study compared with 84 % improvement in Törrönen *et al.* ^(13)^.

Half of the total fibre in the BC was soluble, according to the Finnish food composition database^(39)^. Soluble fibre in the BC might have slightly affected the postprandial responses by increasing the viscosity in the gastrointestinal track and thus reducing the absorption of glucose^(40)^. However, soluble fibre cannot solely explain the beneficial effects of blackcurrant on postprandial glycaemia because blackcurrant nectar and blackcurrant extract with low fibre content also previously induced beneficial effects on the postprandial glucose and insulin responses^(11,13,15)^.

Anthocyanins and proanthocyanidins are the most abundant polyphenols in blackcurrant and may have contributed to the attenuated glycaemic effect (induced mainly by sucrose in the present study) by inhibiting the activity of *α*-glucosidase^(18)^, and thus reducing sucrose digestion and the subsequent absorption from the intestine. Blackcurrant extracts rich in anthocyanins inhibited the activity of *α*-glucosidase *in vitro* ^(41,42)^ but attenuated the postprandial glucose response only slightly after a high-carbohydrate meal while decreasing the postprandial insulin response^(15)^.

Previously, postprandial glycaemia induced by free glucose and fructose was also attenuated by the ingestion of blackcurrant^(13)^, referring to other mechanisms in addition to or instead of inhibition of *α*-glucosidase. Polyphenol-induced inhibition of glucose absorption via GLUT from the small intestine might play a mechanistic role in reducing the postprandial responses^(18,19)^. In a recent *in vitro* study on the potential mechanisms for the reduction of postprandial glycaemia by a blackcurrant extract, glucose uptake and sugar transport were modulated by some minor phenolic compounds rather than anthocyanins^(42)^. At present, the key mechanisms in the regulation of postprandial glucose metabolism by blackcurrants or other berries are not clear, and further studies are needed.

The BCP – also containing 75 g of blackcurrants – reinforced the effects observed by the BC. Particularly, the BCP attenuated the elevation in glucose and insulin concentrations and the subsequent decline in the concentrations. The effect of the BCP was clearly caused by the PB to which the BC was added. Indeed, the PB produced the steadiest postprandial glucose, insulin and NEFA responses. There was a slight difference between the eating times of the test products. However, the difference was in practice only 3–12 s, which is unlikely to make a difference in the postprandial effects of the test products. The somewhat longer eating time for the BCP and PB than for the SW and BC was very likely explained by the larger portion size and the solid state of the BCP and the PB, respectively. The structure of the PB was so rigid that it was not possible to measure the viscosity of the product using a rotary viscometer (Table 1). The dense structure of the PB might explain the steadiest postprandial responses via the delayed absorption of the carbohydrates due to retarded gastric emptying^(43)^. It is of note that the PB contained also stabilising additives, as described below, that may well have had an impact on the postprandial glycaemia. Apart from making the portion size bigger, adding BC to the PB made the BCP fluffier and more palatable. When the structure changed to a less rigid form, this most probably increased the postprandial glucose and insulin concentrations for the BCP as compared with the PB. Quinoa alone is unlikely to have influenced the postprandial responses for the BCP and PB because the amount of quinoa used for the ingested product portion was only 3 g.

The PB contained fat from rapeseed oil and soluble fibres from the stabilising additives, in addition to the fermented quinoa base. The higher content of fat – and thus the higher energy content – may partly explain the retarded effects of the BCP and PB, as compared with the SW and BC. Fat in the ingested meal is known to slow down the rate of gastric emptying^(44,45)^, thus delaying the delivery of carbohydrates to the small intestine for digestion and absorption. *α*-Cyclodextrin contributed mainly to the soluble fibre content of the PB. Intake of *α*-cyclodextrin reduced postprandial glucose concentrations after a high-carbohydrate meal when the origin of available carbohydrates was starch^(46)^. *α*-Cyclodextrin appears to inhibit the effect of pancreatic amylase^(47)^, thus impairing the digestion of starch to absorbable sugar units. However, the effect of *α*-cyclodextrin in lowering the postprandial glucose response induced by sucrose intake was not clearly detected^(48)^.

Along with the modified glycaemia, NEFA concentrations also showed an attenuated increase after the decreased glucose levels. We have previously demonstrated that 150 g of BC is able to attenuate the increase of NEFA in late postprandial phase along with attenuated glycaemic effect when compared with SW^(11)^. Postprandial and longer-term elevation of NEFA concentration may play a role in causing insulin resistance and low-grade inflammation^(49)^. We have also shown^(6)^ that the sharp and continuous increases and decreases in postprandial glycaemia and NEFA levels were related to increased postprandial epinephrine and norepinephrine levels that, in a 12-week long-term intervention with refined cereals, induced increase in low-grade inflammatory markers. Moreover, oral glucose load with 75 g of glucose has previously been shown to increase various inflammation markers postprandially^(33,50,51)^. It has been proposed that the postprandial glycaemic excursions might be one of the key factors within the development in chronic low-grade inflammation^(4,52)^. However, the evidence is somewhat controversial, and the ingestion of 50 g of sucrose or glucose has not always led to increase in inflammatory markers postprandially^(53)^. In line with this, in our present study, we did not see any acute inflammatory effects measured by hs-CRP and using 31 g of available carbohydrates consisting of sucrose, glucose and fructose in the SW, BC, BCP or PC. Previous postprandial studies with raspberries^(54)^ and lyophilised black raspberries consumed with high-fat high-energy meals^(55)^ decreased, however, blood levels of IL-6, had inconsistent effects on TNF-*α* and no effect on hs-CRP. Furthermore, blackcurrant juice with a high-fat meal did not have any effect on postprandial inflammation measured by inflammation markers TNF-*α* and IL-1*β* ^(56)^. Blackcurrant and reasonable amounts of blackcurrant-containing products as such have not been studied regarding postprandial inflammation to the best of our knowledge.

Low amounts of blackcurrant in our present results are, however, most likely not a reason for not showing an effect on inflammatory markers. Instead, we speculate that the volunteers in the present study represent a healthy population with reasonably healthy lifestyles and have no deterioration in their health that would cause metabolic defects. In line with this, the postprandial inflammatory response, measured by hs-CRP in the present study, was not seen even with the SW. Previous studies showing the effect on postprandial inflammation or its alleviation have studied populations at risk of chronic diseases, with chronic diseases or with high-fat diets^(51,54,55)^. Thus, our results should be interpreted with caution, as over half of the volunteers had hs-CRP levels under the detection limit at least in some of the measurements.

One of the main strengths of the present study was that we were able to show the benefits of blackcurrant for postprandial glycaemia with a smaller portion than before. Thus, the added evidence on the benefits of berry consumption with a more consumer-friendly portion may encourage the use of berries in everyday life. These results are easily comparable with the previous studies using the same design, which enables us to add to the relevant scientific understanding on the benefits of berries with varying amounts consumed. Furthermore and unexpected to us, we produced a PB that induced very low glycaemic and insulinaemic responses. When mixed with blackcurrants to gain more pleasant structure and taste, this innovative product would be ideal for prediabetics to balance postprandial glycaemia.

Naturally, there are also weaknesses in our study. With the present study design, we cannot distinguish the possible impact of *α*-cyclodextrin from the effect of fat and the structure of the PB. Thus, further studies are necessary to elucidate the individual effects of the added ingredients in the BCP. Furthermore, to distinguish the possible impacts of dietary fibre from that of the polyphenols, further studies should use, for example, white currants as a reference for blackcurrants. Another weakness is that, despite the open recruitment for both males and females, we were able to recruit mainly female volunteers in the study. This is very typical when volunteers are recruited with open calls – females are more willing to participate in studies concerning healthy foods and food products and to commit for the whole study period. Thus, the results from the present and from our previous studies can be generalised mainly to apply to healthy females.

As a conclusion, blackcurrant, as a 75 g portion and containing all the inherent components of the berry, alleviated sugar-induced excursions and lowered postprandial glycaemia and insulinaemia, as well as balanced NEFA level. These effects were reinforced with a new PB, containing fermented quinoa combined with blackcurrants, which encourages the development of blackcurrant-containing palatable and healthy products.
